# Supplementary material for: Impact of Early Nutritional Intervention During Cancer Treatment on Dietary Intakes and Cardiometabolic Health in Children and Adolescents
Source: Cancers (Basel). 2025 Jan 6;17(1):157. doi: 10.3390/cancers17010157 (PMC11719478; doi:10.3390/cancers17010157)
Supplement: Supplementary file 1 [file cancers-17-00157-s001.zip › cancers-3375821-supplementary.pdf]

Supplementary Table S1. Definitions of cardiometabolic risk factors

Cardiometabolic complications	Age	Criteria				References
**High blood pressure**		**Normal BP**	**Elevated BP**	**Stage 1 HTN**	**Stage 2 HTN**	
	Children (1-13 y)	<90th percentile based on sex, age and height.	≥90th to <95th percentile based on sex, age and height or 120/80 mm Hg to <95th percentile based on sex, age and height (whichever is lower).	≥95th to <95th + 12 mm Hg or 130/80 to 139/89 mm Hg (whichever is lower).	≥95th + 12 mm Hg or ≥140/90 mm Hg (whichever is lower).	[65]
	Adolescents (≥ 13 y) and adults	<120/<80 mm Hg	120/<80 to 129/<80 mm Hg	130/80 to 139/89 mm Hg	≥140/90 mm Hg	[65, 70]
**Blood lipid abnormalities**		**Low**	**Acceptable**	**Borderline**	**High**	[69]
	Children and adolescents	**TC (mmol/L)**				
		-	<4.40	4.40 – 5.17	≥5.18	
		**LDL-C (mmol/L)**				
		-	<2.85	2.85 – 3.35	≥3.36	
		**HDL-C (mmol/L)**				
		<1.03	>1.16	1.03 – 1.16	-	
**High HbA1c**			**Prediabetes**	**Diabetes**		[66]
	Children, adolescents and adults	**HbA1c (%)**	5.7 – 6.4	≥6.5		
**Obesity**		**Underweight**	**Normal**	**Overweight**	**Obese**	[68]
	Children (2 – <5 y)	**BMI-for-age**				
		<-2.0 z-score (<3rd percentile)	-2.0 – 2.0 z-score (3rd – 97th percentile)	>2.0 – 3.0 z-score (>97th – 99.9th percentile)	>3.0 z-score (>99.9th percentile)	
	Children and adolescents	<-2.0 z-score (<3rd percentile)	-2.0 – 1.0 z-score	>1.0 – 2.0 z-score	>2.0 z-score (>97th percentile)	

(≥5 – 19 y)		(3rd – 85th percentile)	(85th – 97th percentile)

y: years, BP: blood pressure, HTN: Hypertension, TC: Total Cholesterol, LDL-C: Low-Density Lipoprotein-Cholesterol, TG: Triglycerides, HDL-C: High-Density Lipoprotein-Cholesterol, HbA1c: Glycosylated Hemoglobin, BMI: Body-Mass-Index.

Supplementary Table S2. Comparison of dietary intakes at end-of-study assessment

	Children[1]			Adolescents[2]		
	Control group n=44	Intervention group n=30	*p*-value[3]	Control group n=33	Intervention group n=15	*p*-value[4]
**Energy**						
kcal						
Mean ± SD	1809 ± 507	1671 ± 361	0.202	2247 ± 777	1936 ± 713	0.194
Median (min – max)	1792 (810 – 2941)	1673 (1074 – 2622)		2100 (1236 – 4370)	1803 (1007 – 3351)	
kcal/kg						
Mean ± SD	76 ± 28.9	70.8 ± 25.3	0.210	33.9 ± 13.2	30.6 ± 12.1	0.421
Median (min – max)	75.3 (25.2 – 160.7)	71.0 (25.1 – 116.5)		34 (18.5 – 49.4)	31.5 (14.2 – 54.6)	
**Fat**						
g/kg						
Mean ± SD	2.8 ± 1.3	2.8 ± 1.2	0.497	1.3 ± 0.7	1.3 ± 0.6	0.958
Median (min – max)	2.7 (0.9 – 5.8)	2.7 (0.8 – 5.5)		1.3 (0.3 – 2.9)	1.3 (0.4 – 2.2)	
% Energy						
Mean ± SD	32.0 ± 7.1	34.6 ± 6.7	0.063	33.3 ± 8.6	37 ± 9.7	0.185
Median (min – max)	31.5 (18.5 – 47.7)	33.4 (19.5 – 50.6)		34 (18.5 – 49.4)	36.6 (14.9 – 52.2)	
n (%)						
> AMDR	13 (29.5)	13 (43.3)	0.159	13 (39.4)	10 (66.7)	0.210
= AMDR	24 (54.5)	16 (53.3)		14 (42.4)	4 (26.7)	
< AMDR	32 (72.7)	1 (3.3)		6 (18.2)	1 (6.7)	
**Carbohydrates**						
g/kg						
Mean ± SD	10.2 ± 4.2	8.9 ± 3.2	0.075	4.2 ± 1.5	3.5 ± 1.7	0.220
Median (min – max)	10.2 (3.4 – 25.1)	8.9 (2.8 – 15.8)		3.6 (1.9 – 8.3)	3.2 (1.5 – 7.5)	
% Energy						
Mean ± SD	53.9 ± 8.9	51 ± 8.7	0.083	50.6 ± 10.5	46.2 ± 10.3	0.220
Median (min – max)	53.8 (22 – 72.2)	49.8 (29.9 – 70.5)		51.8 (30.5 – 72.9)	45.7 (27.1 – 63.4)	
n (%)						
> AMDR	5 (11.4)	2 (6.7)	0.229	2 (6.1)	0 (0)	0.702
= AMDR	34 (77.3)	20 (66.7)		20 (60.6)	9 (60)	
< AMDR	5 (11.4)	8 (26.7)		11 (33.3)	6 (40)	
**Proteins**						
g/kg						
Mean ± SD	2.7 ± 1.0	2.9 ± 1.5	0.299	1.4 ± 0.7	1.3 ± 0.5	0.688
Median (min – max)	2.8 (0.8 – 4.8)	2.9 (0.6 – 6.0)		1.1 (0.4 – 3.2)	1.3 (0.4 – 2.1)	

% Energy						
Mean ± SD	14.7 ± 3.6	16.6 ± 4.5	0.086	16.1 ± 4.6	17.6 ± 5.1	0.295
Median (min – max)	14.5 (8.7 – 24.2)	16.4 (8.8 – 25.7)		15.3 (9.6 – 32.6)	15.8 (10.2 – 27)	
% RDA						
Mean ± SD	289.8 ± 109.3	302.6 ± 147.5	0.343	165.1 ± 83	148.4 ± 56.9	0.483
Median (min – max)	295.6 (87.6 – 506.6)	303.7 (58.1 – 607.2)		131.6 (48.9 – 381.8)	139.5 (52 – 250)	
n (%)						
> RDA	42 (95.5)	29 (96.7)	1.000	29 (87.9)	12 (80)	0.662
< RDA	2 (4.5)	1 (3.3)		4 (12.1)	3 (20)	
**Dietary fiber**						
g/1000 kcal						
Mean ± SD	9 ± 3	9.8 ± 3.7	0.299	7.7 ± 2.3	7.9 ± 2.3	0.732
Median (min – max)	8.8 (4.4 – 19.5)	10.2 (4.1 – 19.3)		7.9 (3.9 – 13.7)	8.0 (3.7 – 11)	
% AI						
Mean ± SD	63 ± 23.4	65.3 ± 27.9	0.703	51.6 ± 20.2	51.4 ± 25.9	0.972
Median (min – max)	63.7 (18.8 – 113.6)	61.3 (25.8 – 112.6)		47.4 (26 – 127.5)	37.2 (17.2 – 99.2)	
n (%)						
> AI	4 (9.1)	2 (6.7)	1.000	1 (3)	0 (0)	1.000
< AI	40 (90.9)	28 (93.3)		32 (97)	15 (100)	
**Sodium**						
mg/1000 kcal						
Mean ± SD	1241.9 ± 419.2	1438.9 ± 389.9	**0.045**	1513.5 ± 655.6	1365.6 ± 421.5	0.429
Median (min – max)	1237.5 (456.7 – 2088.1)	1422.7 (653.4 – 2325)		1520.9 (446.9 – 2920.5)	1401.6 (595.3 – 2330.4)	
% UL						
Mean ± SD	115.3 ± 60.0	126.4 ± 49.2	0.403	146.4 ± 76.5	115.1 ± 48	0.093
Median (min – max)	98.8 (43.3 – 304.1)	116.2 (59.6 – 237.3)		136.9 (34.8 – 311.1)	103.9 (26.1 – 209.2)	
n (%)						
> UL	22 (50)	19 (63.3)	0.342	21 (63.6)	10 (66.7)	1.000
< UL	22 (50)	11 (36.7)		12 (36.4)	5 (33.3)	
**Calcium**						
mg/1000 kcal						
Mean ± SD	457.9 ± 213.0	559.0 ± 233.6	0.058	398.2 ± 170.8	524.4 ± 259.2	0.051
Median (min – max)	442.8 (139.8 – 1218.9)	525.9 (224.0 – 1206.6)		357.9 (127.4 – 744.5)	523.3 (124.0 – 1086.3)	
% RDA						
Mean ± SD	79.1 ± 41.1	93.3 ± 48.5	0.180	84.1 ± 58.6	80.8 ± 48.3	0.846
Median (min – max)	70.8 (16.5 – 183.0)	89.3 (29.0 – 209.8)		71.2 (13.4 – 260.6)	65.2 (9.6 – 157.8)	
n (%)						
> RDA	14 (31.8)	13 (43.3)	0.336	14 (42.4)	6 (40)	1.000

< RDA	30 (68.2)	17 (56.7)		19 (57.6)	9 (60)	
**Vitamin C**						
mg/1000 kcal						0.416
Mean ± SD	66.2 ± 42.0	69.7 ± 51.9	0.761	50.9 ± 46	40.3 ± 27.3	
Median (min – max)	62.3 (1.7 – 170.7)	54.3 (1.8 – 191.0)		39.4 (0.8 – 183)	44.4 (5.1 – 95.9)	
% RDA						0.607
Mean ± SD	406.2 ± 262.7	443.9 ± 351.8	0.619	148.8 ± 130.2	128 ± 125	
Median (min – max)	387.7 (15.7 – 1123.5)	356.5 (7.6 – 1156.2)		148.5 (1.9 – 530.6)	106.4 (22.9 – 511.5)	
n (%)						1.000
> RDA	39 (88.6)	24 (80.0)	0.336	19 (57.6)	8 (53.3)	
< RDA	5 (11.4)	6 (20.0)		14 (42.4)	7 (46.7)	
**Vitamin D**						
µg/1000 kcal						
Mean ± SD	2.3 ± 2.1	2.4 ± 1.5	0.757	2.1 ± 1.8	3.0 ± 2.9	0.319
Median (min – max)	1.9 (0.0 – 9.9)	2.2 (0.2 – 5.4)		1.9 (0.2 – 8.9)	2.4 (0.1 – 9.3)	
% RDA						
Mean ± SD	27.8 ± 25.8	26.8 ± 17.1	0.854	31.9 ± 23.7	41.8 ± 40.7	0.393
Median (min – max)	23.1 (0.2 – 101.3)	24.4 (3.1 – 69.1)		32.4 (1.4 – 81.4)	32.6 (1.3 – 119.8)	
n (%)						
> RDA	1 (2.3)	0 (0)	1.000	0 (0)	2 (13.3)	0.093
< RDA	43 (97.7)	30 (100)		33 (100)	13 (86.7)	

Dietary data were collected using 24-hour recall at the end-of-study (EOS) assessment. Comparison between the control group and intervention group according to age was conducted using Pearson Chi-square (categorical variables), Student's *t*-test (continuous variables).

[1]Participants aged <10 years at cancer diagnosis.

[2]Participants aged ≥10 years old at cancer diagnosis.

[3]*P*-value for the comparison between children of the control group and children of the intervention group.

[4]*P*-value for the comparison between adolescents of the control group and adolescents of the intervention group.

SD: Standard Deviation; EER: Estimated Energy Requirement; AMDR: Acceptable Macronutrient Distribution Range; RDA: Recommended Dietary Allowance; AI: Adequate Intake; UL: Tolerable Upper Intake Level.

Supplementary Table S3. Comparison of diet quality scores at end-of-study assessment

	Children[1]			Adolescents[2]		
	**Control group n=44**	**Intervention group n=30**	***p*-value[3]**	**Control group n=33**	**Intervention group n=15**	***p*-value[4]**
**Diet Quality Index (DQI)[5]**						
Score of 100 points						
Mean ± SD	56.3 ± 11.9	54.4 ± 10.9	0.492	48.1 ± 11.7	46.7 ± 11.7	0.728
Median (min – max)	57.0 (32.5 – 74.0)	55.8 (30.5 – 77.0)		42.5 (32.5 – 77.5)	42.5 (32.5 – 77.5)	
**Healthy Diet Index (HDI)[6]**						
Score of 7 points						
Mean ± SD	3.1 ± 1.5	2.5 ± 1.3	0.073	2.8 ± 1.3	2.9 ± 1.1	0.783
Median (min – max)	3.0 (0 – 7)	2.0 (0 – 6)		3.0 (1 – 6)	3.0 (1 – 5)	
n (%)						
Low	27 (61.4)	24 (80.0)	1.00	24 (72.7)	11 (73.3)	1.00
Medium	14 (31.8)	5 (16.7)		8 (24.2)	4 (26.7)	
High	3 (6.8)	1 (3.3)		1 (3.0)	0 (0.0)	

Dietary quality scores were calculated from nutritional data collected using 24-hour recall at the end-of-study (EOS) assessment. Comparison between the control group and the intervention group according to age was conducted using Student's *t*-test and Fisher's exact test (level of adherence).

[1]Participants aged <10 years at cancer diagnosis.

[2]Participants aged ≥10 years old at cancer diagnosis.

[3]*P*-value for the comparison between children of the control group and children of the intervention group.

[4]*P*-value for the comparison between adolescents of the control group and adolescents of the intervention group.

[5]DQI is a continuous score with a maximum of 100 points [61], where a higher total score indicates a better quality diet.

[6]HDI is based on the sum of 9 components for which a value of +1 is assigned when intake is within the recommended range or otherwise as 0, generating a score ranging from 0 to 9. The level of adherence is determined as low (0-3), medium (4-6), and high (≥7) [61].

SD: Standard Deviation.

Supplementary Table S4. Comparison of anthropometric measures at end-of-study assessment

	Children[1]			Adolescents[2]		
	**Control group**	**Intervention group**	***p*-value[3]**	**Control group**	**Intervention group**	***p*-value[4]**
**Weight**	**n=44**	**n=30**		**n=33**	**n=15**	
kg						
Mean ± SD	25.9 ± 9.3	26.1 ± 8.5	0.936	69.6 ± 18.8	70.7 ± 34.2	0.914
Median (min – max)	23.7 (13.2 – 66.2)	24 (14.1 – 53.8)		65.5 (37.6 – 127.0)	59.3 (29.2 – 166.9)	
z-score						
Mean ± SD	0.10 ± 1.03	0.24 ± 1.08	0.578	0.51 ± 1.32	0.86 ± 2.05	0.476
Median (min – max)	0.15 (-1.78 – 2.39)	0.06 (-1.62 – 2.87)		0.39 (-1.76 – 3.59)	0.41 (-1.98 – 6.18)	
**Height**	**n=43**	**n=30**		**n=33**	**n=15**	
cm						
Mean ± SD	124.1 ± 14	122 ± 12.6	0.513	169.5 ± 9.9	164.1 ± 14.4	0.138
Median (min – max)	122.5 (97.2 – 177.4)	122.3 (97 – 143.2)		171.2 (137.0 – 184.5)	166.9 (129.8 – 179.5)	
z-score						
Mean ± SD	-0.11 ± 0.97	-0.29 ± 0.80	0.410	-0.04 ± 0.90	-0.15 ± 1.20	0.717
Median (min – max)	-0.14 (-1.78 – 2.78)	-0.08 (-1.96 – 1.01)		0.01 (-2.40 – 1.41)	-0.19 (-3.26 – 2.17)	
**BMI**	**n=43**	**n=30**		**n=33**	**n=15**	
kg/m²						
Mean ± SD	16.5 ± 2.3	17.1 ± 3.1	0.313	24.1 ± 5.4	25.2 ± 9.0	0.582
Median (min – max)	16 (13 – 22.5)	16.5 (14.2 – 28.8)		22.6 (18 – 39.2)	22.0 (16.7 – 51.8)	
z-score						
Mean ± SD	0.22 ± 1.08	0.54 ± 1.09	0.221	0.58 ± 1.32	1.04 ± 1.77	0.322
Median (min – max)	0.19 (-2.09 – 2.41)	0.46 (-0.96 – 3.04)		0.62 (-1.48 – 3.43)	0.79 (-1.68 – 5.62)	
**Waist circumference**	**n=39**	**n=26**		**n=30**	**n=13**	
cm						
Mean ± SD	62.8 ± 9	63.2 ± 10.2	0.864	89.7 ± 13.8	88.8 ± 19.1	0.868
Median (min – max)	59.8 (49.6 – 88.8)	61 (51 – 97.3)		86.7 (71.1 – 128.7)	85.4 (61 – 123.4)	
z-score						
Mean ± SD	0.61 ± 0.70	0.53 ± 0.94	0.687	0.88 ± 0.66	0.87 ± 1.03	0.947
Median (min – max)	0.61 (-1.19 – 1.77)	0.61 (-1.79 – 2.15)		0.85 (-0.18 – 2.22)	0.95 (-1.62 – 2.14)	
**MUAC**	**n=42**	**n=28**		**n=33**	**n=14**	

cm						
Mean ± SD	20.4 ± 2.8	20.1 ± 3.8	0.693	30.8 ± 4.5	29.8 ± 6.4	0.574
Median (min – max)	19.5 (16.4 – 29.4)	19.7 (10.7 – 31.8)		29.7 (23.9 – 44.8)	28.4 (20.2 – 40.7)	
z-score						
Mean ± SD	0.81 ± 0.83	0.41 ± 1.38	0.135	0.78 ± 1	0.67 ± 1.32	0.743
Median (min – max)	0.68 (-0.63 – 2.20)	0.53 (-4.95 – 2.58)		0.87 (-1.12 – 3.05)	0.67 (-1.28 – 2.61)	
**TSFT**	**n=40**	**n=27**		**n=31**	**n=14**	
mm						
Mean ± SD	12.1 ± 6.7	11 ± 3.5	0.415	18.8 ± 10.8	17 ± 6.9	0.574
Median (min – max)	10.1 (4.4 – 36.2)	10.2 (5.8 – 20.4)		15.8 (5.0 – 57.0)	15.2 (9 – 33.8)	
z-score						
Mean ± SD	-0.05 ± 1.40	0.30 ± 0.91	0.219	0.85 ± 1.13	0.55 ± 0.90	0.379
Median (min – max)	-0.12 (-2.66 – 3.47)	0.29 (-1.73 – 2.14)		0.77 (-1.41 – 3.46)	0.69 (-1.29 – 2.23)	
**SSFT**	**n=38**	**n=26**		**n=30**	**n=13**	
mm						
Mean ± SD	7.6 ± 6.4	6.4 ± 2.7	0.386	15.9 ± 10.2	12.9 ± 7.4	0.344
Median (min – max)	5.4 (3.3 – 38.1)	5.5 (3.0 – 14.3)		12.7 (5.8 – 41.0)	10.8 (5.2 – 32.0)	
z-score						
Mean ± SD	-0.16 ± 1.01	0.10 ± 1.18	0.350	0.47 ± 1.21	0.33 ± 0.97	0.697
Median (min – max)	-0.26 (-2.79 – 2.70)	0.16 (-2.64 – 2.30)		0.37 (-1.52 – 2.35)	0.50 (-1.25 – 2.11)	

Data on anthropometric measures were collected at the end-of-study (EOS) assessment. Comparison between the control group and the intervention group according to age was conducted using Student's *t*-test.

[1]Participants aged <10 years at cancer diagnosis.

[2]Participants aged ≥10 years old at cancer diagnosis.

[3]*P*-value for the comparison between children of the control group and children of the intervention group.

[4]*P*-value for the comparison between adolescents of the control group and adolescents of the intervention group.

BMI: Body Mass Index; MUAC: Mid-Upper Arm Circumference; TSFT: Triceps Skinfold Thickness; SSFT: Subscapular Skinfold Thickness; SD: Standard Deviation.

Supplementary Table S5. Comparison of blood pressure and biochemical parameters at end-of-study assessment

	Children[1]			Adolescents[2]		
	**Control group**	**Intervention group**	***p*-value[3]**	**Control group**	**Intervention group**	***p*-value[4]**
**Blood pressure**						
Systolic						
mmHg	**n=44**	**n=28**		**n=32**	**n=14**	
Mean ± SD	97 ± 10	99 ± 7	0.294	113 ± 14	111 ± 11	0.678
Median (min – max)	97 (76 – 118)	99 (88 – 118)		113 (73 – 136)	112 (96 – 134)	
z-score	**n=44**	**n=28**		**n=32**	**n=11**	
Mean ± SD	0.15 ± 0.87	0.17 ± 0.80	0.934	-0.18 ± 1.14	-0.38 ± 0.63	0.478
Median (min – max)	0.12 (-1.41 – 2.33)	-0.01 (-1.41 – 1.69)		-0.04 (-2.33 – 2.33)	-0.23 (-1.36 – 0.91)	
Diastolic						
mmHg	**n=44**	**n=28**		**n=32**	**n=14**	
Mean ± SD	57 ± 6	61 ± 10	0.112	66 ± 8	61 ± 8	0.071
Median (min – max)	58 (42 – 69)	58 (47 – 98)		66 (49 – 84)	61 (45 – 73)	
z-score	**n=44**	**n=28**		**n=32**	**n=11**	
Mean ± SD	-0.01 ± 0.51	0.28 ± 1.03	0.171	-0.15 ± 0.78	-0.53 ± 0.71	0.170
Median (min – max)	-0.02 (-0.81 – 1.48)	0.01 (-1.04 – 3.92)		-0.24 (-1.75 – 1.75)	-0.60 (-2.24 – 0.33)	
n (%)	**n=44**	**n=28**		**n=32**	**n=14**	
Normal BP	37 (84.1)	22 (78.6)	0.754	19 (59.4)	11 (78.6)	0.179
High BP	7 (15.9)	6 (21.4)		13 (40.6)	3 (21.4)	
**Blood lipids**						
TC (mmol/L)	**n=44**	**n=28**		**n=33**	**n=14**	
Mean ± SD	4.06 ± 0.62	4.19 ± 0.95	0.484	4.26 ± 1.02	4.25 ± 1.50	0.984
Median (min – max)	3.98 (2.94 – 5.58)	4.01 (3.10 – 8.07)		4.17 (2.27 – 6.79)	3.98 (2.29 – 8.53)	
n (%)	**n=44**	**n=28**		**n=33**	**n=14**	
Normal value	41 (93.2)	25 (89.3)	0.672	27 (81.8)	12 (85.7)	1.000
Low value	3 (6.8)	3 (10.7)		6 (18.2)	2 (14.3)	
LDL-C (mmol/L)	**n=44**	**n=27**		**n=33**	**n=14**	
Mean ± SD	2.35 ± 0.60	2.36 ± 0.97	0.936	2.61 ± 0.88	2.56 ± 1.43	0.884
Median (min – max)	2.34 (1.26 – 3.74)	2.24 (1.21 – 6.38)		2.59 (0.60 – 5.04)	2.25 (0.84 – 6.80)	
n (%)	**n=44**	**n=27**		**n=33**	**n=14**	
Normal value	40 (90.9)	26 (96.3)	0.643	28 (84.8)	11 (78.6)	0.679

Low value	4 (9.1)	1 (3.7)		5 (15.2)	3 (21.4)	
HDL-C (mmol/L)	**n=44**	**n=27**		**n=33**	**n=14**	
Mean ± SD	1.41 ± 0.24	1.41 ± 0.29	0.905	1.15 ± 0.26	1.11 ± 0.23	0.669
Median (min – max)	1.37 (0.95 – 2.01)	1.43 (0.74 – 1.85)		1.16 (0.68 – 1.85)	1.18 (0.77 – 1.54)	
n (%)	**n=44**	**n=27**		**n=33**	**n=14**	
Normal value	43 (97.7)	24 (88.9)	0.290	19 (57.6)	9 (64.3)	0.462
Low value	1 (2.3)	3 (11.1)		14 (42.4)	5 (35.7)	
**Glucose metabolism**						
HbA1c (%)	**n=40**	**n=22**		**n=31**	**n=11**	
Mean ± SD	5.11 ± 0.31	5.18 ± 0.36	0.462	5.19 ± 0.27	5.01 ± 0.34	0.082
Median (min – max)	5.10 (4.30 – 5.70)	5.20 (4.40 – 5.80)		5.20 (4.70 – 6.00)	5.10 (4.50 – 5.40)	
n (%)	**n=40**	**n=22**		**n=31**	**n=11**	
Normal value	34 (85)	17 (77.3)	0.499	27 (87.1)	11 (100)	0.330
High value	6 (15)	5 (22.7)		4 (12.9)	0 (0)	
**Vitamin D**						
(nmol/L)	**n=42**	**n=21**		**n=32**	**n=14**	
Mean ± SD	76.5 ± 20.3	67.4 ± 30.5	0.165	61.9 ± 25	62.3 ± 28	0.958
Median (min – max)	73.5 (23.0 – 132.8)	65.3 (14.5 – 151.3)		64.5 (15 – 117)	59.2 (28 – 126.5)	
n (%)	**n=42**	**n=21**		**n=32**	**n=14**	
Sufficiency	21 (48.8)	11 (52.4)	0.604	20 (62.5)	10 (71.4)	0.783
Insufficiency	20 (46.5)	8 (38.1)		10 (31.3)	4 (28.6)	
Deficiency	1 (2.3)	2 (9.5)		2 (6.3)	0 (0)	

Data on cardiometabolic health indicators were collected at the end-of-study assessment. Comparison between the control group and the intervention group according to age was conducted using Pearson Chi-square (categorical variables) and Student's *t*-test (continuous variables).

[1]Participants aged <10 years at cancer diagnosis.

[2]Participants aged ≥10 years old at cancer diagnosis.

[3]*P*-value for the comparison between children of the control group and children of the intervention group.

[4]*P*-value for the comparison between adolescents of the control group and adolescents of the intervention group.

HbA1C: Glycosylated Hemoglobin; TC: Total Cholesterol; HDL-C: High-Density Lipoprotein-Cholesterol; LDL-C: Low-Density Lipoprotein-Cholesterol; SD: Standard Deviation.
